# Effect of Holder pasteurization and UV-C irradiation on bacteriophage titres in human milk

**DOI:** 10.1093/femsle/fnad057

**Published:** 2023-06-16

**Authors:** Lisa F Stinson, Donna T Geddes, Lucy L Furfaro

**Affiliations:** School of Molecular Sciences, The University of Western Australia, Perth 6009, Australia; School of Molecular Sciences, The University of Western Australia, Perth 6009, Australia; Division of Obstetrics and Gynaecology, Medical School, The University of Western Australia, Perth 6009, Australia; Women and Infants Research Foundation, Subiaco 6008, Australia

**Keywords:** bacteriophage, donor milk, Holder pasteurization, human milk, UV-C

## Abstract

Human milk is the optimal nutrition source for infants and contains a complex mix of bioactive compounds and microorganisms. When unavailable, pasteurized donor milk may be provided, particularly to preterm infants. Holder pasteurization (HP) is typically implemented in human milk banks to prevent pathogen transmission. Given the impact of heat on milk bioactives, ultraviolet-C irradiation (UV-C) is an alternative being explored and has demonstrated effective bactericidal activity. In addition to bacteria, milk contains viruses, including primarily bacteriophages (phages) and which likely influence the developing bacterial microbiome of infants. However, the effect of pasteurization on human milk phages is unknown. This study assessed the effect of HP and UV-C on titres of exogenous bacteriophages inoculated into human milk. Ten donor human milk samples were tested in parallel with water controls. Milk samples or water controls were inoculated to a final concentration of 1 × 10^4^ PFU/mL (±1 log) each of a thermotolerant *Escherichia coli* phage (T4) and a thermosensitive *Staphylococcus aureus* phage (BYJ20) and subjected to HP and UV-C treatments. UV-C inactivated both phages within milk and water controls, however, HP was ineffective against the thermotolerant T4 phages. Initial data suggest that UV-C treatment may eliminate phage with potential to affect preterm infant gut colonization. Further studies should extend this to other phages.

## Teaser

Human milk pasteurization methods impact bacteriophage titres, with potential consequences for donor milk fed infants.

## Introduction

Holder pasteurization (HP) is used by human milk banks globally to eradicate bacteria from donor human milk samples. This technique, which involves heating donor milk to 62.5°C for 30 minutes, results in a 5-log_10_ reduction of commensal and potentially pathogenic bacteria, including *Escherichia coli, Staphylococcus epidermidis, Enterobacter cloacae*, vegetative cells of *Bacillus cereus*, and *Staphylococcus aureus* (Czank et al. 2009). While the ability of HP to reduce bacterial numbers in human milk is well characterized, the effect of this treatment on non-bacterial microbes in milk remains understudied with research focused on human pathogens (Friis and Andersen 1982, Orloff et al. 1993, Walker et al. 2020, Pitino et al. 2021). The effect of HP on milk bacteriophages (phages) is currently unknown.

Phages are bacterial viruses that are often highly specific to their bacterial host. Both the human milk virome and the infant gut virome are dominated by phages (Lim et al. 2015, Pannaraj et al. 2018), with phage diversity being highest in early life (Lim et al. 2015). The infant gut phage-ome is initially dominated by prophage, which is derived from maternal sources (including milk) and induced within the infant gut (Duranti et al. 2017, Liang et al. 2020, Shamash and Maurice 2022). Phages modulate human health largely via their effects on the bacterial microbiota (Weinbauer and Rassoulzadegan 2004, Dahlman et al. 2021). However, despite the role of phages in human health, there is only limited data describing phage dynamics in early life. This is a critical gap, given that early life is a key window for assembly and maturation of gut bacterial populations, with life-long consequences for host health (Stewart et al. 2018, Stiemsma and Michels 2018, Stinson 2020). The study of the infant gut phage-ome is limited by the large portion of uncharacterized viral diversity within infant gut samples, with 70% of infant viral taxa unable to be matched to gut viral databases (Shah et al. 2023). Given the predatory relationships between lytic phages and their bacterial hosts, phages may influence early life bacterial colonization patterns.

It is not known whether pasteurization of donor milk affects the viability of phages. Importantly, given that phages can be thermotolerant or thermosensitive (Jonczyk et al. 2011), HP may have variable effects on different phage populations. Interestingly, data from the dairy industry suggest that milk itself may intrinsically provide thermal protection to phages. In one study of *Leuconostoc* phages, a 1-minute heat treatment at 70°C on the test phage P808 resulted in a low phage reduction (1 log unit) when the phage was suspended in bovine milk, but a high phage reduction (4 log units) when the phage was suspended in water (Atamer et al. 2011). These data suggest that milk provides thermal protection to some phages, which may indicate that HP may be ineffective against phage.

In addition to HP, ultraviolet C (UV-C) irradiation has been proposed as a novel form of treatment for donor human milk (Christen et al. 2013a). UV-C treatment reduces milk bacterial numbers to a similar extent as HP, but has the additional benefit of preserving many bioactive components of milk, which are destroyed by heating (Christen et al. 2013a, b). While the impact of UV-C irradiation on phage titres in human milk is currently unknown, evidence from the dairy industry suggests that UV-C light treatment may be more effective at destroying phages in bovine milk than heat pasteurization (Atamer et al. 2013).

Here, we aimed to characterize the effect of HP and UV-C irradiation on titres of thermosensitive and thermotolerant exogenous phages in human milk.

## Methodology

### Participants and sample collection

Mothers of infants aged 0–12 months were invited to donate milk samples (*n* = 11; 200–800 mL) for this study. Participants expressed and stored the samples in their home freezers prior to donation (−20°C, maximum 9 months storage). This study was approved by the University of Western Australia’s Human Research Ethics Committee (RA/4/1/2369) and all participants provided written informed consent.

### Milk characteristics

Total fat, total protein, and total solids were measured in each human milk sample prior to inoculation and pasteurization. Total fat was measured using the validated creamatocrit method (Lucas et al. 1978, Du et al. 2017). Total protein was measured in skim milk in duplicate using the Bradford method (Bradford 1976). Total solids content was measured by pre-drying samples on a boiling water bath, followed by evaporation and in a drying oven at a temperature of 102°C (Standardization, 2018).

### Bacteriophage preparation

Two phages were selected based on their thermostability characteristics and included thermo-resistant dsDNA phage *Escherichia coli* phage T4 (select as a reference phage) and thermo-sensitive dsDNA *Staphylococcus aureus* phage BYJ20 (laboratory phage isolated from wastewater, selected due to the high abundance and prevalence of *Staphylococcus* in human milk). The propagating hosts included *Escherichia coli* B (reference strain) and *Staphylococcus aureus* SSCC 61935 (clinical isolate) and were cultured overnight in Tryptic Soy Broth (TSB; Becton Dickinson, USA) at 37°C, 250 rpm. High titre stocks of 10^8^ PFU/mL bacteriophages were propagated as per standard overlay methods described previously (Furfaro et al. 2020) and purified using centrifugal filtration (Amicon 100 KDa, Merck, Germany) in Sodium Magnesium (SM) buffer (pH 7.5) (Bonilla et al. 2016).

### Inoculation of human milk samples and water controls

**Figure 1.**
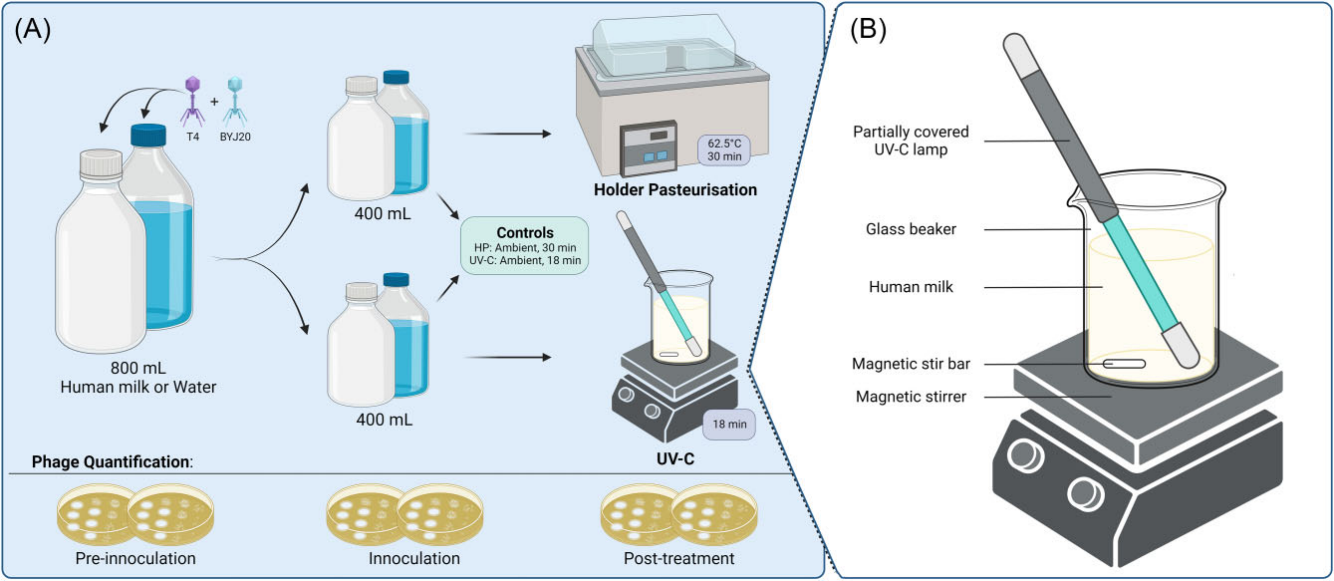
An overview of the experimental approach (A) and details of the UV-C irradiation equipment set up (B).

For each experiment (*n* = 10), 800 mL of human milk was used. Given that this volume was not always obtainable from a single donor, milk samples were pooled from two donors for one experiment. These samples were combined in a sterile 1-L Schott bottle and gently mixed by inverting. The remaining nine experiments consisted of milk from single donors. Samples were inoculated with *Escherichia coli* phage T4 and *Staphylococcus aureus* phage BYJ20 to a final concentration of 1 × 10^4^ PFU/mL (±1 log) each and again gently mixed (Fig. 1a).

To assess any potential thermal/UV-C protection that milk may provide for phages, a water control was included alongside each experimental batch. A volume of 800 mL of MilliQ water was inoculated with phages as above and processed in an identical manner to the milk samples.

### HP and UV-C irradiation

Each milk sample (*n* = 10) and water control (*n* = 10) was divided into two 400 mL aliquots. One aliquot was Holder pasteurized by heating to 62.5°C in a water bath for 30 minutes. Temperature was gauged using a thermal probe (Thermocouple Thermometer, Delta Ohm, Italy). UV-C irradiation was performed as previously described (Christen et al. 2013a). Briefly, samples were placed into a sterile 500-mL beaker in a sterile laminar flow hood. A germicidal UV-C lamp (95% of UV-C output at 253.7 nm; Infralight Pty Ltd, Helensburgh, NSW, Australia) was placed diagonally into the beaker, so that the uncovered portion of the lamp was submerged in the sample (Fig. 1b). Samples were stirred with a magnetic stir bar (500 rpm) to create a low velocity laminar flow vortex of milk around the lamp, ensuring all of the sample was exposed to the light throughout the duration of the treatment (18 minutes). UV-C radiance was measured for each experiment using a UV-C light meter (X9_11µ_ UV-C Meter, Gigahertz-Optik, Türkenfeld, Germany). UV-C dosage (fluence) was calculated as radiance (W/m^2^) × exposure time (seconds). Following HP or UV-C irradiation, samples were immediately aliquoted and phage titre determined via plaque assays.

Given that human milk has antimicrobial properties (Lonnerdal 2003), there was the possibility that phage titres may be affected by the milk *per se*, regardless of pasteurization method. To account for this, phage inoculated untreated aliquots of milk and water were stored at room temperature for the duration of each experiment and then titred to assess surviving number of phages (non-treatment controls).

### Bacteriophage quantification

Bacteriophage activity was quantified using the spot test method as described previously with modifications (Furfaro et al. 2020). Briefly, double agar overlays were prepared using Tryptic soy agar (TSA; Becton Dickinson, USA), whereby 4 mL of molten TSA (0.5%) and 250 µL of propagating host bacteria (*Escherichia coli* B or *Staphylococcus aureus* clinical isolate SSCC 61935) were layered over a solid TSA plate. Neat and dilutions (in SM buffer) of the experimental milk and water samples were spotted onto the overlays in triplicate 10 µL spots. Plates were incubated at 37°C under atmospheric conditions overnight and individual plaques quantified after 24 h. All results were standardized based on the quantification of bacteriophages resulting from immediate inoculation prior to treatment for each sample (original titre post-inoculation).

### Statistical analysis

Statistical analyses were performed using IBM SPSS Statistics for Windows, version 28.0.1.0 (IBM Corporation, USA). Descriptive statistics revealed non-normal distribution resulting in use of the non-parametric independent samples Kruskal–Wallis test. Bonferroni correction was used to account for multiple tests and significance was set at an alpha value of 0.05.

## Results

### Milk characteristics

Total fat, total protein, and total solids for the tested samples were within expected ranges for mature human milk (Table 1; Geddes et al. 2017).

**Table 1.** Total fat, total protein, and total solids of the human milk samples used for pasteurization.

Sample	Total fat (g/L)	Total protein (g/L)	Total solids (g/L)
1	38.2	14.46	111.45
2	34.1	10.36	149.14
3	42.4	6.82	106.05
4	35.9	7.62	173.52
5	43.6	5.69	109.02
6	47.1	3.33	114.37
7	44.7	2.43	107.04
8	82.6	4.18	111.00
9	46.5	4.93	97.54
10	28.8	7.67	106.82

### Holder pasteurization

**Figure 2.**
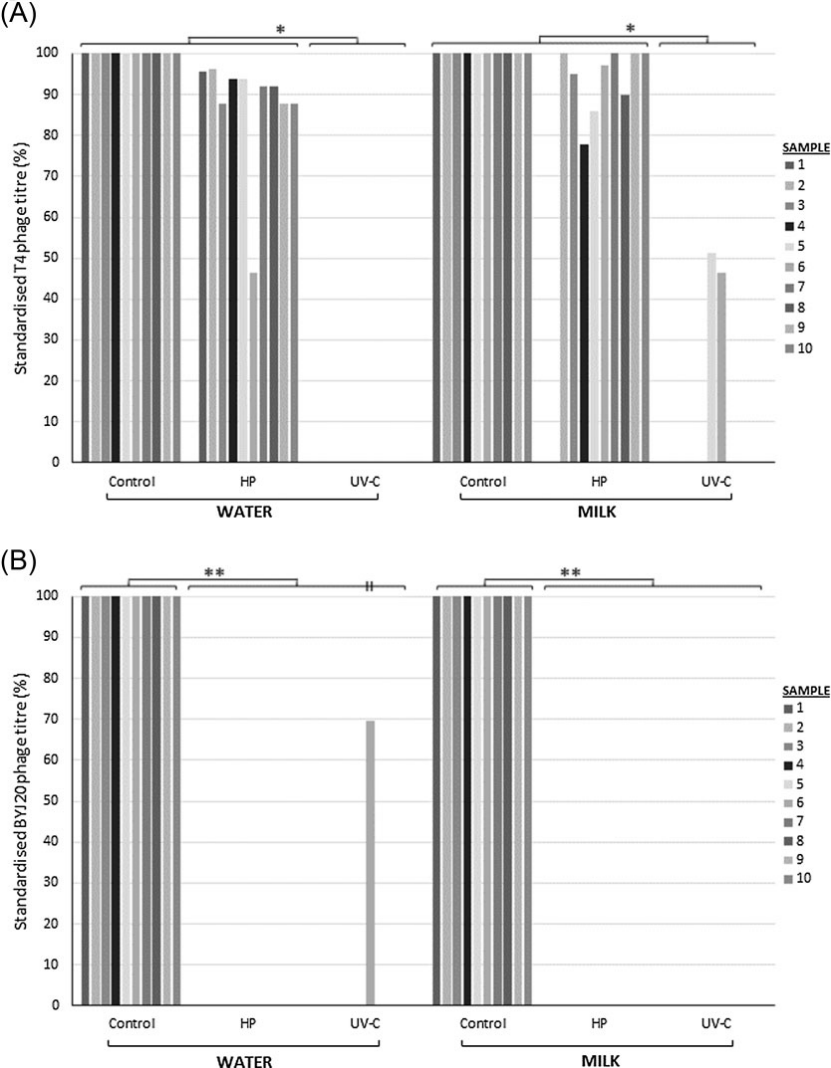
The effect of HP and UV-C treatment (UV-C) compared to non-treatment controls (Control) on: (A) *E. coli* phage T4 and (B) *S. aureus* phage BYJ20 in both water (Water) and human milk (Milk). A total of 10 samples were tested and are represented as individual columns for each variable. Significant differences between HP and UV-C treatment of phage T4 (*P < 0.003). BYJ20 phage showed significant difference between Control and both treatment groups (UV-C and HP) (**P < 0.01). Overall, no difference was observed between water and human milk samples.

HP had little impact on the thermo-resistant phage T4 with titres within 1 log of the inoculated untreated milk (*P* = 1.000; Fig. 2) and minimal difference in T4 titre was observed between the non-treatment control and HP-treated water samples (*P* = 0.274). Additionally, plaques were more defined and larger for T4 after HP compared to pre-treatment. In contrast, *Staphylococcal* phage BYJ20 (thermo-sensitive) was consistently inactivated by HP methods (*P* < 0.001).

### UV-C irradiation

An average UV-C dose of 1879.2 J/m^2^ was achieved during the 18-minute treatment time (SD ± 1058 J/m^2^). UV-C irradiation displayed complete consistent inactivation across almost all treatments of both T4 and BYJ20 phages (*P* < 0.001; Fig. 2), noting T4 phage withstood UV-C inactivation in two human milk samples (∼50% reduction in titre) and BYJ20 phage in one water sample (30% reduction in titre). There was no observed major difference between phage titres corresponding to suspension in water or human milk.

### Non-treatment controls

To test the impact of the milk itself, non-treatment controls were included in the study. There was no significant difference between the controls and the inoculated pre-treatment groups (*P* > 0.05). Similarly, non-inoculated milk was tested for endogenous phage activity against the propagating hosts used in this study and only one of the 10 milk samples had observed phage activity against *E. coli* B host. This was purified and plaque production confirmed, however, the low titre present in the raw milk (single plaque on neat) did not impact the results obtained.

## Discussion

Numerous studies have highlighted the abundance of bacteriophages present in human milk (Pannaraj et al. 2018, Liang et al. 2020, Mohandas and Pannaraj 2020, Dinleyici et al. 2021), demonstrating the need for assessment of the impact that pasteurization techniques have on these types of viruses. Here, we demonstrate that phage are differentially impacted by UV-C irradiation and HP of human milk. HP was able to eradicate thermosensitive *S. aureus* phage (BYJ20), however, thermotolerant *E. coli* phage (T4) remained viable with minimal reduction in titre. In contrast, UV-C treatment eradicated both T4 and BYJ20 phages in 8/10 samples, with no observable activity following treatment.

Removal of viral agents in human milk may be beneficial when considering pathogenic viruses such as Zika virus, cytomegalovirus, and human immunodeficiency virus (Black 1996, Michie and Gilmour 2001, Blohm et al. 2018); however, the importance of bacteriophages in the early life microbiome is not well understood. A recent analysis of the human milk virome found that 92% of all human milk samples tested had viruses detected and further observed differences in bacteriophages predominance with respect to lactation period, preterm birth, mode of delivery, and infant birth weight (Dinleyici et al. 2021). For the safety of preparing donor milk, UV-C treatment is a promising method for inactivation of viruses such as bacteriophages, however, given their abundance in healthy human milk (Pannaraj et al. 2018, Liang et al. 2020, Mohandas and Pannaraj 2020, Dinleyici et al. 2021), one might speculate of their role in the early stages of microbiome development. Indeed, vertical transmission of Bifidobacteria phages has been demonstrated from mother to infant via human milk (Duranti et al. 2017). If bacteriophages play a role in shaping the early life bacterial microbiome, their inactivation by commonly used milk bank pasteurization methods may impact early microbiome establishment in donor milk fed infants. In particular, donor milk is frequently fed to preterm infants, whose gut microbiomes are known to vary from those of full term infants (Aguilar-Lopez et al. 2021). These infants are particularly vulnerable to bacterial infections, such as *E. coli*, leading to necrotizing enterocolitis. Faecal filtrate transplants suggest that bacteriophages may play a role in protection from gut bacterial infections (Ott et al. 2017, Brunse et al. 2021). However, the current evidence on bacteriophage populations in human milk and the early life gut is sparse. More work is needed to understand phage–bacteria dynamics in infants.

Given that infant gut phage profiles are determined by breastfeeding (Liang et al. 2020), and that gut phages modulate the bacterial microbiota (Liang et al. 2020) and exert direct host effects (Gorski et al. 2018, Fluckiger et al. 2020), we suggest that destruction of milk phages by pasteurization may impact infant health. Indeed, eradication of thermo-sensitive phages in Holder pasteurized donor milk may contribute to the differences seen in the gut microbiome and health outcomes of donor milk fed and mother’s own milk fed infants (Parra-Llorca et al. 2018, Pineiro-Ramos et al. 2021). The present study highlights that different milk processing techniques can have effects on phage populations. Understanding these effects is an important first step towards examining endogenous milk phages in future studies to further contextualize our findings.

While milk phages may act to maintain a balanced infant gut microbiome, phages may also pose a risk to vulnerable preterm infants. In fact, transfer of antimicrobial genes via human milk has been acknowledged (Das et al. 2019), potentially implicating milk phages as mobile genetic elements and a potential source of gene transfer among milk taxa such as *Staphylococcus aureus* (temperate phages in particular). Therefore, there may be incidences where eradication is warranted. Further, pasteurization may result in induction of temperate phages. Bacterial stress response activation can lead to the induction of prophages, with UV a well-known inducing agent (Klaenhammer and McKay 1976). Therefore, the effects of UV treatment on endogenous bacteria and subsequent prophage induction in human milk is an important topic for future study. Our study provides vital information to enable the field to assess the various parameters that impact the milk microbiome and the potential effects of processing techniques on bacteriophage activity.

While previous work in bovine milk suggested that milk provides thermal protection to certain phage (Atamer et al. 2011), this did not appear to be the case here, potentially due to differences in the physiochemical composition of human and bovine milk. Overall, human milk did not appear to influence the stability of the phages against each treatment as the results from the water controls were not statistically different to those of milk. Milk derivatives such as skim milk have been assessed as microbial cryopreservation agents previously, however, with variable outcomes (Cody et al. 2008). It has been suggested that skim milk may affect the fatty acid content of the bacterial cell membranes, which may, in turn, change the viscosity of membranes and help to stabilize cell enzymes (Guo et al. 2020). However, in the present study, where whole milk was used, this did not appear to be the case in stress conditions such as heat (HP) and UV-C irradiation. Similarly, this result is promising for UV-C methods, highlighting penetration was not an issue in this study, with the continuous movement and direct UV-C exposure sufficient to eradicate both phages within human milk, which is a high opacity solution.

UV-C irradiation of human milk has previously been shown to inactivate the eukaryotic virus cytomegalovirus, whilst retaining the bioactivity of the milk itself (Lloyd et al. 2016). Our study further supports this result, with UV-C treatment observed to inactivate both phages used, while HP was only able to inactivate the thermosensitive phage (BYJ20). Whilst milk bioactivity was not assessed in this study, previous studies (Christen et al. 2013b, Lloyd et al. 2016, Martysiak-Żurowska et al. 2017, Almutawif et al. 2019) have demonstrated minimal impact of UV-C on milk proteins and bioactive components, including secretory IgA, lactoferrin, and lysozyme. As a result, UV-C treated milk has been shown to induce better weight gain and intestinal health in preterm piglets (Li et al. 2017). This makes UV-C treatment a promising novel pasteurization technique for donor human milk. However, we note that data from studies of human (Martysiak-Żurowska et al. 2017) and animal (Urgu-Ozturk 2022) milk suggest that UV-C irradiation may result in lipid oxidation, which may produce undesirable organoleptic effects. Further optimization of UV-C radiance and treatment time may identify a UV-C treatment protocol that eliminates microorganisms while minimizing oxidation and other unwanted effects.

The impact of UV-C on bacteriophages has been well studied in general, with early reports of effective use of UV-C to remove *Streptococcus lactis* bacteriophages from commercial dairy plants (Greene and Babel 1948). Using a lamp source above rather than submerged within the filtrate, they found that the wattage of the lamp, distance from the sample, and concentration of bacteriophages influenced the time required to inactivate the bacteriophages. In comparison to our methods, the recommended time to destroy all bacteriophages at a titre of 10^3^ PFU/mL of phages, 3 inches from the lamp source (279 µW) was 21 minutes (Greene and Babel 1948). Our study has the benefit of the UV-C lamp being submerged and the steady flow of the solution allowing the entire sample to be directly exposed to the UV-C irradiation, which has resulted in complete inactivation in 18 minutes (average UV-C dosage 1879.2 J/m^2^). The titre of phages used in the current study was moderate (10^4^ PFU/mL); however, despite the high likelihood of other endogenous phages present naturally within the milk (targeting different hosts), we still observed inactivation of the phages present. To assess the scalability of this approach, future studies may require assessment of various titres.

Here, we describe the impact of UV-C compared to HP on phages in human milk. We show that while UV-C irradiation efficiently destroys both thermotolerant and thermosensitive phage, HP is only effective against thermosensitive phage. Our results have broad implications given the potentially beneficial role phages may play in the infant gut microbiome. While exogenous phage were tested in this proof-of-concept study, the effect of UV-C and HP treatment on the endogenous human milk ‘phageome’ should be assessed to better understand the impact of donor milk treatment on the developing infant gut microbiome. In particular, if human milk phage play a role in protecting preterm infants from neonatal necrotizing enterocolitis and other bacterial infections, the impact of donor milk pasteurization techniques on milk phage must be considered.
